# Differential Location and Distribution of Hepatic Immune Cells

**DOI:** 10.3390/cells6040048

**Published:** 2017-12-07

**Authors:** Maria Alice Freitas-Lopes, Kassiana Mafra, Bruna A. David, Raquel Carvalho-Gontijo, Gustavo B. Menezes

**Affiliations:** 1 Center for Gastrointestinal Biology, Departamento de Morfologia, Instituto de Ciências Biológicas, Universidade Federal de Minas Gerais, Belo Horizonte, Minas Gerais 31270-901, Brazil; licefl95@gmail.com (M.A.F.-L.); kassiana93@gmail.com (K.M.); 2 Calvin, Phoebe and Joan Snyder Institute for Chronic Diseases, Department of Physiology and Pharmacology, University of Calgary. Calgary, AB T2N 1N4, Canada; brunaraujodavid@gmail.com

**Keywords:** liver, hepatocytes, hepatic leukocytes, immune response

## Abstract

The liver is one of the main organs in the body, performing several metabolic and immunological functions that are indispensable to the organism. The liver is strategically positioned in the abdominal cavity between the intestine and the systemic circulation. Due to its location, the liver is continually exposed to nutritional insults, microbiota products from the intestinal tract, and to toxic substances. Hepatocytes are the major functional constituents of the hepatic lobes, and perform most of the liver’s secretory and synthesizing functions, although another important cell population sustains the vitality of the organ: the hepatic immune cells. Liver immune cells play a fundamental role in host immune responses and exquisite mechanisms are necessary to govern the density and the location of the different hepatic leukocytes. Here we discuss the location of these pivotal cells within the different liver compartments, and how their frequency and tissular location can dictate the fate of liver immune responses.

## 1. Introduction

The liver is one of the largest organs in the body, weighing up to 1.5 kg (3.3 lb.) in adults [1]. Interestingly, although this can constitute only ~2.5% of body weight, the liver receives around 25% of the cardiac output [2]. The liver is located in the upper right portion of the abdominal cavity, beneath the diaphragm and on top of the stomach, right kidney, and intestines. The human liver has of two main lobes, which are divided in eight segments (four each lobe). The segments are then microscopically divided in hepatic lobules, which may have anatomic variations between different species. Blood enters into the liver to circulate within the lobules through liver microvessels, while bile, produced and secreted by hepatocytes, flows in the opposite direction. Bile drains via several bile canaliculi that connect with larger ducts to ultimately form the common hepatic duct. The common hepatic duct transports bile to the gallbladder, and finally to its final destination: the duodenum.

The liver has a unique hemodynamic scheme. Blood from the spleen, pancreas, and gastrointestinal circulation reaches the liver via the portal vein together with arterial input from the hepatic artery. It is estimated that ~80% of the blood volume arises from the portal circulation, while the remaining ~20% originates from arterial flow [3,4]. Once they enter the liver, both portal vein and hepatic artery will branch into smaller segments to irrigate several liver lobules through the hepatic portal spaces. Blood will then slowly flow within the sinusoids, being later drained out of the liver by the centrilobular vein via the hepatic vein. This single vascular architecture together with slow blood flow creates an intimate relationship between the systemic circulation and liver cells. The high permeability of capillary endothelium to water, macromolecules, and solutes is explained by the presence of special transporting systems, including channels, vesicles, diaphragms, and fenestrae. In fact, liver sinusoids are one of the most permeable microvessels in the body, and millions of large fenestrae (>100 nm) can be found along the sinusoidal surface [5]. Lining their lumen, there is a specialized subtype of endothelial cell named LSECs (liver sinusoidal endothelial cells). LSECs comprise around ~20% of liver cells [6] and are located at the interface between hepatic microcirculation and hepatocytes. On the luminal side, LSECs continuously survey blood from the gastrointestinal tract, exerting a close relationship with resident liver macrophages (Kupffer cells) and all leukocytes that are in the circulation or those that constantly patrol liver vessels (including monocytes, NK, and NKT cells). On the other side (facing the Disse Space), LSECs interact with hepatic stellate cells (or Ito cells) and hepatocytes. This is crucial for liver metabolism since LSECs are a permeable barrier that mediates the exchange, active uptake, and degradation of circulating molecules [7]. LSECs also possess a high endocytic capacity, and numerous endocytic vesicles can be found under homeostatic conditions in their cytoplasm. It is well accepted that LSECs may perform effective uptake of a wide variety of substances from the blood by receptor-mediated endocytosis [8]. Therefore, considering the absence of a regular basal lamina together with the presence of fenestrae, LSECs are different and unique from any other type of endothelial cell in the body.

**Figure 1:**
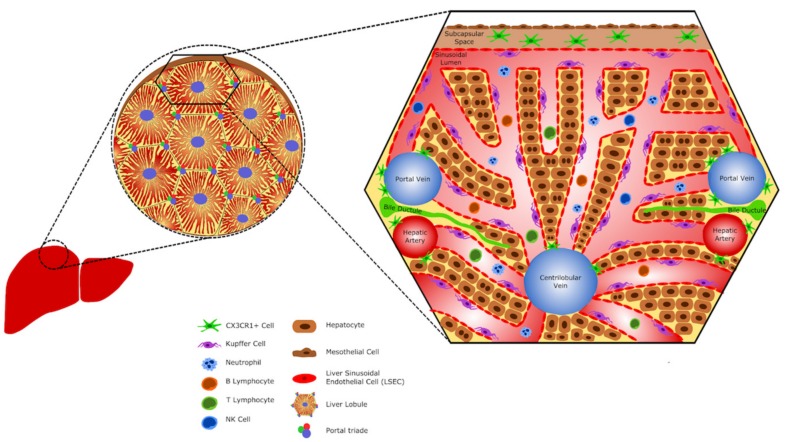
**The hepatic immune cells.** Schematic representation showing hepatic cells and their location. The liver harbors a large population of immune cells. Dendritic cells (CX3CR1^+^ cells) can be found in the subcapsular space and surround large vessels, as the centrilobular vein and the portal triade vessels (portal vein and hepatic artery). Kupffer cells are the liver resident macrophages and constitute the largest population of hepatic immune cells. They can be found within sinusoids, in contact with endothelial cells. Neutrophils, B lymphocytes, T lymphocytes, and NK cells eventually circulate in the sinusoids.

Considering that liver hemodynamic features and vascular architecture create a niche for blood surveillance, it is not surprising that [9] the hepatic environment harbors one of the largest populations of immune cells in the body. Virtually, subsets of all leukocytes and resident phagocytes can be found within the liver even under homeostatic conditions. Strikingly, these cells are not randomly distributed throughout the tissues; instead, they are strategically located within the different hepatic compartments (intravascular and subcapsular niches; discussed below), while a large population of these immune cells can be also found patrolling the sinusoidal lumen (Figure 1). In addition, liver immune cell population can be rapidly and dramatically changed during inflammation, and this can be associated with the pathogenesis of several diseases. In this review, we will discuss how the differential tissular location of liver immune cells may influence their function during homeostasis and disease.

## 2. Immune System Ontogeny and the Correlation with the Fetal Liver

**Figure 2:**
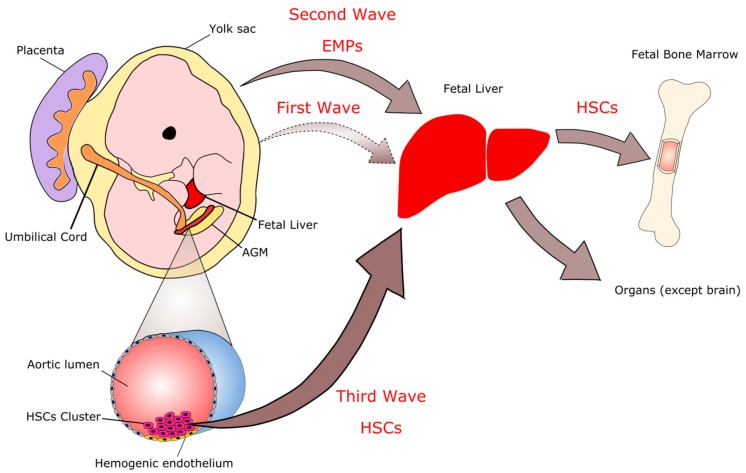
**Immune system ontogeny.** The colonization of immune cells in the liver occurs in three waves. The first wave originates in blood islets within the yolk sac on embryonic day E7.0. These cells are transient, being replaced by the cells of the second and third waves. The second wave also begins in the yolk sac as erythromyeloid progenitors (EMPs). The third wave gives rise to hematopoietic stem cells (HSCs) from the hemogenic endothelium at the aorta/gonada/mesonephros (AGM) region. EMPs and HSCs seed in the liver and this organ becomes the main place of hematopoiesis in the embryo. From the liver, the immune cells colonize other organs in the body, including the bone marrow, which replaces the hematopoietic function of the liver at the end of gestation and becomes the hematopoietic organ of the adult.

The liver harbors different immune cell populations that are established during the embryonic period of life. The ontogeny of fetal macrophages occurs in successive and overlapping waves that arise from both extra- and intra-embryonic sites, leading to the sequential acquisition of myeloid, erythroid, and lymphoid lineage potentials (Figure 2). The yolk sac is the first hematopoietic organ where macrophages develop in mammals [8,9,10,11,12] and several studies have reported that macrophages arise in embryos before the generation of the first hematopoietic stem cells (HSCs). In mouse embryos, the first wave is termed primitive hematopoiesis and develops from the posterior plate mesoderm in the blood islands of the extra-embryonic yolk sac around E7.5, giving rise to primitive erythroblasts, megakaryocytes, and primitive macrophages [9,13,14,15]. These cells are derived from c-Kit^lo^ CD41^lo^ precursors, which are also dependent on the expression of the transcription factor PU.1 [13,16].

Between E8.0 and E8.5, the heart is formed and the fetal cardiovascular system is established and connected with the vitelline and umbilical vessels [17]. At this time, a second wave of hematopoietic progenitors occurs, called erythro-myeloid precursors (EMPs). They arise from the yolk sac hemogenic endothelium [13,16]. These progenitors (EMPs) are phenotypically defined as c-Kit^+^ AA4.1^+^ (CD93^+^) CD41^+^ VE-cadherin^+^ CD16/32^+^ (FCgII and FCgIII receptors) CD45^lo^ [18,19] and exhibit erythroid and broad myeloid—but not lymphoid—potential [20]. EMP-derived hematopoiesis is sufficient to support survival of HSC deficient embryos until birth [21]. In addition, EMPs emerge in a Runx1-dependant endothelial-to-hematopoietic transition [22]. The number of EMPs in the yolk sac peaks between E9.5 and E10.5, and they seed the fetal liver as soon as E9 [23,24]. These cells will expand and differentiate into multiple lineages, including fetal macrophages, which will colonize the liver giving rise to Kupffer cells (KCs) and colonize to other organs, including the fetal lung [25]. Yolk-sac EMPs express the gene encoding the transcription factor c-Myb (Myb) [26], but their commitment and differentiation into the myeloid fate is unaltered in c-Myb-deficient embryos, although their erythroid potential is blocked [25,27,28]. Therefore, c-Myb is required for the commitment and differentiation of EMPs into the erythroid fate [29] but is dispensable for myeloid differentiation.

Almost concomitant with the emergence of the late EMPs, a new wave of hematopoietic progenitors arises from the intraembryonic hemogenic endothelium, which begins with the generation of immature HSCs in the para-aortic splanchnopleura region and proceeds to give rise to fetal HSCs in the aorta, gonads, and mesonephros regions at E10.5 [30], as well as the umbilical and vitelline arteries [31]. These precursors migrate to the fetal liver, where they expand and differentiate from E12.5 until definitive hematopoiesis begins to shift to the bone marrow. HSCs colonize the embryonic bone marrow at E15, and active hematopoiesis starts at E17 [24,32]. Fetal and adult HSCs require c-Myb for their self-renewal and maintenance, and loss of c-Myb expression leads to rapid HSC-derived hematopoiesis failure [27,33,34]. In addition, HSCs also require the transmembrane receptor Notch1 for their emergence, in contrast to EMPs, as Notch1^−/−^ embryos have normal numbers of hematopoietic progenitor cells in the yolk sac but very few in the body of the embryo [35].

At 12 days of gestation, the number of liver macrophages with the ability to engulf blood cells rapidly increases and most of them are attached to the endothelial cells in the sinusoid. From E12.5, the fetal liver becomes the major hematopoietic organ within the embryo and contains progenitors of different origins and varied potentials, which together will give rise to the emergent immune system. The hepatic hematopoiesis becomes most prominent from 16 to 18 days of gestation, whereas it decreases in the perinatal period and disappears within a week after birth.

## 3. Differential Location of Immune Cells throughout the Liver

### 3.1. Phagocytes

#### 3.1.1. Macrophages and Monocytes

Hepatic macrophages were first observed in 1876 by Karl Wilhelm von Kupffer, who described them as an integral part of the sinusoid endothelium and were initially called “Sternzellen” (star cells) [7]. In 1898, after several years of research, Tadeusz Browicz correctly identified them as macrophages, and they received the name of Kupffer-Browicz cells, also known as Kupffer cells (KCs). The liver comprises the largest population of resident macrophages in the body representing ~80–90% of total fixed macrophages and 35% of the liver non-parenchymal cells in normal adult mice [36]. Different from other organs where the resident macrophages are located in the tissue parenchyma, in the liver these cells are inside the sinusoids in direct contact with blood circulation [7,17,37,38]. KCs are adhered to the endothelium and emit extensions into the extravascular space (space of *Dissé*) acting as a bridge between the blood and components of the liver parenchyma. Moreover, the continuous structure formed by KCs and endothelial cells forms the reticuloendothelial system (RES), which serves as the first line of defense against particles and immunorreactive material passing from the gastrointestinal tract via the portal circulation and may be considered the final component in gut barrier function [39].

**Figure 3:**
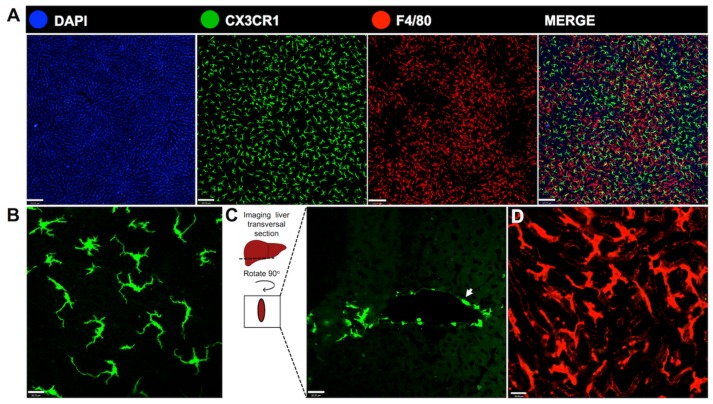
**In vivo visualization of distinct morphological aspects of liver phagocytes.** (**A**) Intravital imaging of CX3CR1^gfp/wt^ reporter mice under homeostatic condition revealed large hepatic cell populations with different spatial distribution: CX3CR1^+^ cells (in green), which is not expressed by liver macrophages and are suggestive of a dendritic cell population, and Kupffer cells stained with anti-F4/80 antibody (in red), merged with DAPI (in blue), a marker for DNA used to identify the nucleus of the cells in the field. Images through the hepatic surface showed that CX3CR1^+^ cells are abundant and uniformly distributed, unlike Kupffer cells; (**B**) a higher magnification of image showing the dendritic morphology of dendritic cells in the capsule; (**C**) transversal section of frozen tissue evidencing the fluorescence of hepatic parenchyma. There are CX3CR1^+^ cells around a large vessel (arrow), with a more circular form and less dendrites; (**D**) numerous Kupffer cells within the liver parenchyma, evidenced by a distinct morphology. Scale bars in (**A**), 120 μm. Scale bars in (**B**–**D**), 26 μm. All images were acquired using an inverted Nikon Eclipse Ti coupled to an A1 scanning head with no modifications. All animal studies were approved by the Animal Care and Use Committee at Universidade Federal de Minas Gerais, Brazil (CEUA 147/2016).

Kupffer cells have the ability to migrate through the tissue against or in favor of blood flow by locomotion at 4 μm/min on average [40], half the speed observed in neutrophils that patrol the liver [41]. However, their speed greatly reduces when these cells perform phagocytosis and many of them lose their ability to move in these conditions [40]. Despite this locomotion ability described under homeostasis conditions, KCs maintain a constant pattern of tissue distribution. Most of them are in the sinusoidal zones, close to the portal spaces, and in smaller numbers in the centrilobular zones [38]. These patterns of location also affect morphology, phagocytic capacity, and the metabolic functions exerted by the cells in these different regions of the hepatic lobules [42,43]. In addition to morphological, functional, and tissue location variations, hepatic macrophages are also a heterogeneous phenotype population. Under homeostatic conditions, two F4/80^+^ Kupffer cell subsets may exist: a CD68^+^ subset with phagocytic activity and a CD11b^+^ subset with cytokine producing capacity [44]. Furthermore, subpopulations of KCs have differential expression of CD11c and major histocompatibility complex class I and class II (MHCI and MHCII) [38]. Interestingly, unlike other organs and tissues such as brain, intestine, lung, kidneys, spleen and skin, hepatic macrophages do not express the fractalkine receptor CX3CR1 in homeostatic conditions (Figure 3) [38,45]. More recently, an important common feature between KC subsets that allows their separation from other resident macrophages is the expression of the gene *Clec4f*, a KC-specific marker [46,47,48].

For many years, it was believed that tissue-resident macrophages are originated and continuously maintained by blood-circulating monocytes, which arose from progenitors in the adult bone marrow (BM). This cellular hierarchy was a foundational concept in the definition of the “mononuclear phagocyte system” (MPS) by Van Furth and colleagues in the 1970s that grouped together promonocytes and their precursors in the BM, monocytes in the peripheral blood, and macrophages in the tissues [49,50]. After the discovery of a common myeloid precursor (macrophage DC precursor-MDPs) of monocytes, macrophages, and dendritic cells, the knowledge of the ontogeny of the cell types within the MPS has dramatically changed [51]. Since then, efforts have been dedicated in deciphering the developmental lineages of monocytes, DCs, and macrophages [52,53,54,55,56]. Currently, the MPS grouped together monocytes, macrophages, dendritic cells, and all their precursors based on morphology, expression of surface markers, functional specialization, and ontogeny [57]. Although monocytes and macrophages belong to the same group, recent ontogeny studies have shown that the emergence, establishment, and maintenance of resident macrophages populations—such as KCs—are independent of circulating monocytes [25,56,58,59,60].

In the fetal liver, macrophages play a scavenger function and support hematopoiesis. These macrophages begin to show peroxidase activity in the nuclear envelope and endoplasmic reticulum after 17 days of gestation [15,61,62], corresponding to KCs in the adult liver [63,64]. Fetal macrophages rapidly expand to transform into KCs in the late stage of ontogeny and after birth. Fetal tissue macrophages also have a high proliferative capacity that is important for their survival in loco and for their colonization from the fetal liver to the other fetal tissues via blood stream. From this point of view, the fetal liver is a central organ for producing and supplying macrophages and their precursors to the other tissues. Although myeloid precursors are found in fetal hematopoiesis, the production of monocytes during the hematopoiesis of the yolk sac is poorly developed. Compared to monocytes originating from bone marrow precursors, the number of peroxidase-positive granules in monocytes originating from yolk sac precursors is significantly lower [9]. In the fetal liver, monocytes increase in number and show an increase in the number of peroxidase-positive granules [15]. In the middle stages of hepatic hematopoiesis, their ultra-structural features resembled those seen during bone marrow hematopoiesis.

Further studies have revealed that the contribution of HSCs to tissue-resident macrophages differs among organs and frequently increases with age. The contribution of HSCs to adult tissue-resident macrophages is minor (<5%) in the brain, liver, and epidermis [26,27,58]. Although small, their contribution increases with age in the lungs, heart and spleen [25,65], and might predominate in gut lamina propria after weaning [66,67]. Partial replacement of tissue-resident macrophages is also observed following γ-irradiation, bone marrow transplantation, or adoptive-transfer experiments [51,68,69] and in macrophage-depletion studies, such as intravenous injection of clodronate-loaded liposomes (CLL) [38]. KCs are also reported to be replaced by bone marrow–derived progenitor cells following, for example, massive death of Kupffer cells in severe experimental infection with *Listeria monocytogenes* [70] and drug induced liver injury. However, different resident macrophages—including KCs, microglia, alveolar macrophages, peritoneal macrophages, and splenic macrophages—have the potential to proliferate and self-renewing [37,59,71,72]. In some cases, tissue-resident macrophages can immediately self-replenish following severe depletion [59,71,73] and exert their functions in the tissue.

Macrophages play a central role in both tissue homeostasis and inflammation, accomplishing essential tissue-specific functions as well as protecting the organism from infection. It is currently believed that the characteristic functions exerted by the different populations of resident macrophages are attributed to three main factors: their exposure to specialized tissue environments [46,74,75], the contribution of distinct embryonic or fetal progenitors to distinct subsets [25,58,76] and the preferential expression of transcription factors [46]. The rapid recognition and bacterial clearance from the blood is a crucial step in the first-line innate immune defense against systemic infection. In liver, the efficient phagocytosis of pathogens is ensured by the strategic location of the KCs and by their different phagocytic mechanisms. One of them is via the complement receptor of the superfamily Ig, named CRIg [77]. In addition to phagocytosis mediated by Fc receptors, KCs recognize bacteria opsonized by the C3b and iC3b complement component via CRIg, which enables the removal of pathogens from circulation [77]. CRIg is also important in the detection and uptake of viral vectors through recognition of C3 complementary components present in viruses [78]. However, the internalization of viral particles is associated with higher rates of KC depletion, compromising host innate immune response and increasing the susceptible to systemic infections [78].

Highlighting the relevance of a rapid removal of bacteria from the circulation in the prevention of systemic infections, recent studies have identified new mechanisms of phagocytosis performed by resident macrophages in the liver. It has been shown that bacteria that reach the liver through arterial blood (fast flow) are rapidly phagocytosed via scavenger receptors when they remained non-opsonized and not bound to platelets [79]. However, bacteria flowing through the venous blood (slow flow) are rapidly opsonized, binding to platelets and being phagocytosed via CRIg [79], elucidating two distinct bacterial clearance pathways. Moreover, scavenger receptors are the main receptor family that mediates a fast-track clearance of bacteria, and phagocytosis of Gram-positive bacteria by KCs may occur even when opsonization with complement is not present [80]. In this case, CRIg on KCs directly binds lipoteichoic acid (LTA) on Gram-positive bacteria, such as *Staphylococcus aureus* and *Listeria monocytogenes*. However, it is not clear if CRIg is relevant in the capture of Gram-negative bacteria [80].

The close proximity of KCs to parenchymal and nonparenchymal cells supports their ability to regulate hepatic function, both in health and disease. In a healthy liver, KCs exhibit a tolerogenic phenotype promoting and maintaining what has been termed “immunological tolerance”: an anti-inflammatory mechanism to limit deleterious tissue injury in infections [81]. This tolerance is necessary to prevent overt immune responses against immunoreactive molecules from the hepatic sinusoids, including gut-derived antigens, and also damage-associated molecular patterns (DAMPs) from dead or dying cells as they are cleared from the circulation in the liver [81,82]. Mechanistically, tolerance in liver can be established by either direct deletion or tolerogenic priming of CD8 T cells [83,84] or by induction of regulatory T-cell responses [85,86]. This function of ensuring immunological tolerance is also related to phagocytosis. Particles removed from circulation can induce tolerogenic T-cell responses in homeostatic conditions, preventing immune diseases in other organs [87]. It is important to emphasize that this induced liver tolerance is directly related to the original KCs with tolerogenic profile (M2-like), which are different from infiltrated monocytes (M1-like) with immunogenic profiles. This means that upon tissue injury, tolerance might be broken [87].

Another important feature of macrophages is the plasticity that allows the adaptation and phenotypic alteration according to environmental changes, which lead to the activation of KCs and their consequent differentiation in M1-like macrophages (classical) and M2-like macrophages (alternative) [88]. Despite the actual value of segregating the diverse macrophage polarization phenotypes under the “M1/M2 category” still being under debate and might be excessively simplistic, in this review, we will still referee to these populations in this way due to didactic reasons. Inflammatory cytokines and microbial products, such as LPS, can induce differentiation of KCs in an M1-like profile [89]. M2-like profile can be induced by IL-4, IL-10, IL-13, IL-33, transforming growth factor (TGF-β), and granulocyte colony-stimulating factor (G-CSF). M1-like macrophages are key effector cells for the elimination of pathogens, virally infected, and cancer cells and produce large amounts of IL-12, IL-23 [90], nitric oxide (NO), and production of ROS [91]. M2-like macrophages, in turn, are usually associated with resolution and tissue repair, being responsible for the production of IL-10, TGF-β, and extracellular matrix components [91]. In fact, the dysregulation of the inflammatory (M1)/tolerogenic (M2) phenotypic balance is an important mechanism governing the pathogenesis of chronic inflammatory diseases, suggesting that strategies restraining macrophage polarization may protect against exacerbated inflammation and thus limit tissue injury. Moreover, activation of M1 KCs to secrete pro-inflammatory mediators is a key event in the initiation of fatty liver diseases. However, tolerogenic M2 KCs are able to induce apoptosis of activated M1 KCs by inhibiting pro-inflammatory signaling and reducing tissue damage [92].

During liver injury, the dynamics of monocytes and macrophages varies according to tissue damage. In mild injuries that lead to moderate or no loss of tissue-resident macrophages, or when few or no blood monocytes are recruited, macrophage repopulation occurs exclusively from the initial endogenous tissue-resident population. The remaining embryonic-derived macrophages have the potential to repopulate themselves locally [59]. In infection models, macrophage repopulation occurs from both local and blood-derived precursors, which ultimately leads to a mosaic ‘macrophage chimera’ situation with mixed macrophage compartments that are of both embryonic and adult origin [78,93]. However, we do not yet fully understand whether the blood-derived cells persist and become fully integrated into the macrophage network, or they are a temporary addition to the endogenous population. Macrophage repopulation occurs from blood monocytes or blood-derived precursors following severe inflammatory injuries that lead to major tissue-resident macrophage loss or the partial suppression of their self-renewal capacities [48,59,71]. In situations of hepatocyte necrosis, KCs may help in recruiting circulating monocytes into the damaged tissue and these infiltrating monocytes are responsible for an increase in tumor necrosis factor-α, and the subsequent proliferation of liver progenitor cells (LPCs) [94]. Recent data on ontogeny and different origins of resident macrophages has raised questions about the possible consequences of the substitution of original macrophages of embryonic origin by macrophages derived from monocytes throughout life and by various tissue lesions. Different groups have demonstrated that the emergency replacement of liver macrophages has acute [38] and long-term [48] consequences. Therefore, understanding the origins, the developmental pathways and the homeostatic processes that regulate tissue-resident macrophages is fundamental to enable the design of future intervention strategies to modulate macrophage functions at specific sites.

#### 3.1.2. Dendritic Cells

Paul Langerhans was the first to describe the dendritic cells (DCs). He characterized the Langerhans cells in the skin, and the term ‘dendritic cell’ was coined by Steinman, Cohn, and Banchereau due to their morphology: they have large nucleus, abundant cytoplasm and dendrites [95,96]. DCs are antigen-presenting cells (APCs) capable of inducing immune and tolerogenic responses in lymphoid and non-lymphoid organs, including the liver. Their location within the liver has been disputed over the years, although it is now well established that hepatic DCs represent a heterogeneous and large population within the liver immune milieu [97].

**Figure 4:**
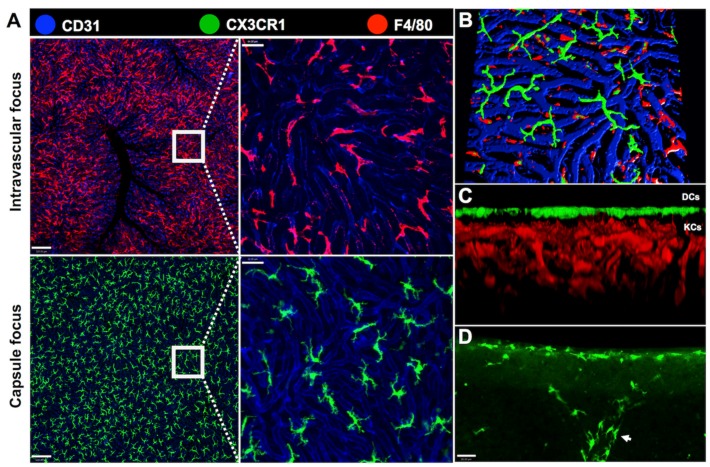
**Distribution of hepatic phagocytes within liver intravascular and extravascular compartments.** Intravital microscopy of CX3CR1^gfp/wt^ reporter mice enables the visualization of liver phagocytes in both intravascular and extravascular compartments. (**A**) Kupffer cells (in red) are observed in an intravascular focus. They are located inside the sinusoids, here stained with andi-CD31 PECAM-1 (in blue). Their location facilitates the capture of circulating bacterial products or intact bacteria by these cells. To image the extravascular cells only, the images were obtained in the capsule focus of the liver. There is an exclusively extravascular population of CX3CR1^+^ cells (in green) that inhabit the liver surface; (**B**,**C**) 3-dimensional reconstructions of liver compartments evidencing the intravascular population (KCs in red) and the extravascular population (DCs in green). The CX3CR1^+^ cells are found especially underneath the liver capsule and are rarely distributed in the parenchyma; (**D**) CX3CR1^+^ cells underneath the liver capsule and around a large vessel (arrow). Scale bars in (**A**), 120, 64, 36 μm. Scale bars in (**D**), 26 μm. All images were acquired using an inverted Nikon Eclipse Ti coupled to an A1 scanning head with no modifications. All animal studies were approved by the Animal Care and Use Committee at Universidade Federal de Minas Gerais, Brazil (CEUA 147/2016).

Hepatic DCs have been described as an interstitial and nonphagocytic cell population residing periportally, around central veins and in the liver capsule [98,99,100]. The DCs located underneath the liver capsule are morphologically different from the ones found around large vessels: capsular DCs are larger and have more dendrites [38]. Unlike KCs, DCs are rarely distributed within the parenchyma and immunohistochemical staining of normal adult livers shows that hepatic DCs express MHC class II. Recent studies have indicated that there is a distinct and large hepatic resident cell population inhabiting the subcapsular space, the CX3CR1^+^ cells. Gene expression analysis between classical splenic DCs and CX3CR1^+^ cells isolated from mice liver classifies them as potential hepatic DCs [38], once they have a high ability to present antigens and lower phagocytic behavior. Nevertheless, it is not clear whether these cells are DCs or a distinct non-KC macrophage population [101]. The presence of a widespread and distinct cell population underneath the mesothelium suggests that the location of these cells may be strategic for sealing and preventing liver exposure to bacteria and hazard substances from the peritoneal cavity into the liver in certain injury contexts, since the liver capsule is in contact with the peritoneal cavity. Therefore, these cells may play an important role not by directly killing pathogens, but presenting antigens and recruiting other phagocytic cell types to the injury site. Once they represent a numerous cell population in the liver, it is essential to study their origin and their role in different contexts (Figure 4).

Dendritic cells are essential to capture, process, and present antigens by interacting with T cells, playing an important role to initiate immune responses. DCs have a distinct role in the liver to maintain a tolerogenic condition due to liver contact to products of digestion, from drug metabolism, microorganism products and intact bacteria [102,103]. The heterogeneous population of DCs in the liver is described to be fully derived from the bone marrow, mainly from common-myeloid precursors (CMPs). Three subsets of hepatic murine DCs (CD19^−^CD11c^+^) are now characterized: lymphoid (CD8α^+^B220^−^CD11b^−^), myeloid (CD8α^−^B220^−^CD11b^+^), and plasmacytoid (B220^+^CD11b^−^). DCs may be also classified into two main subsets that include classical/conventional DCs (cDCs) and plasmacytoid DCs (pDCs) [76]. Murine cDCs express typical myeloid antigens and are typically distinguished as CD11c^+^MHCII^+^. They consist of two types of cells: cDC1s and cDC2s. The cDC1 cells resemble CD8^+^ lymphoid DCs, have migratory capacity and are efficient in presenting cell-associated antigens [55,104]. The cDC2s development in most non-lymphoid organs depends on the presence of FMS-like tyrosine kinase 3 ligand (FLT3L), a factor that enhances global T cell and humoral immunity, and macrophage colony-stimulating factor (M-CSFR). The cDCs also bestow two subsets: CD103^+^CD11b^+^ and CD103^+^CD11b^−^. Another hepatic DCs population, the plasmacytoid DCs, expresses lower levels of MHCII and functions as major producer of type I interferons (IFNs) in response to viral infections. pDCs can be characterized as CD11c^low^ or CD11c^+^CD11b^−^B220^+^Gr-1^+^ in mice [105,106,107,108].

Different subsets of DCs are identified in fetal tissues and are related to adult populations, and they mediate immune responses during gestation [109]. However, neither of the molecules here presented exclusively identify DCs. Many of the surface markers can be used to study other myeloid cell types, such as neutrophils and lymphocytes, especially during inflammation. For example, there are mice KCs that express CD11c in steady state conditions and during hepatic replenishment after clodronate injection [38]. Despite CD11c is considered a classic marker for DCs, it is not very reliable when studying hepatic DCs. Therefore, it is important to effectively excluding these double-cell populations in certain analysis. These immunophenotype strategies are relevant to study the functions of DCs in different types of hepatic injury.

During homeostasis, DCs are tolerogenic and immature. The immature dendritic cells (IDCs) interact with antigens by capturing: they phagocyte particles and direct them to compartments rich in MHCII to form MHCII-peptide complexes [110]. In a context of chronic inflammation, mature DCs have a proinflammatory profile. For example, in a model of murine fibrosis induced by nonalcoholic steatohepatitis (NASH), hepatic CD11c^+^ DCs are able to limit CD8^+^ T cells expansion, produce elevated immune-modulatory cytokines—such as IL-6 and TNFα, but interestingly not IL-10—and activate CD4^+^ T cells [111], hence modulating hepatitis and fibrosis in NASH. In addition, it is well known that hepatic DCs are pivotal in the generation of both innate and adaptive immunity in response to lipopolysaccharide (LPS). In a murine experiment model, Chen et al. isolated hepatic DCs and characterized the expression of toll-like receptor 4 (TLR4) in response to LPS stimulation, cytokine productions and ability to ablate T cells after mice had been injected with plasmid-GM-CSF [112]. Liver DCs had a role in stimulating a regulatory response as expanding CD4^+^Foxp3^+^ T regulatory cells and promoting secretion of IL-27. These data suggest an immunoregulatory role of these cells. Nevertheless, in response to viral infections, hepatic CD103^+^ DCs induce and sustain CD8^+^ T cells activity against hepatotropic antigens in situ [113], opening possibilities to target these cells for strategies like treatments and vaccination.

### 3.2. Granulocytes 

#### 3.2.1. Neutrophils

**Figure 5:**
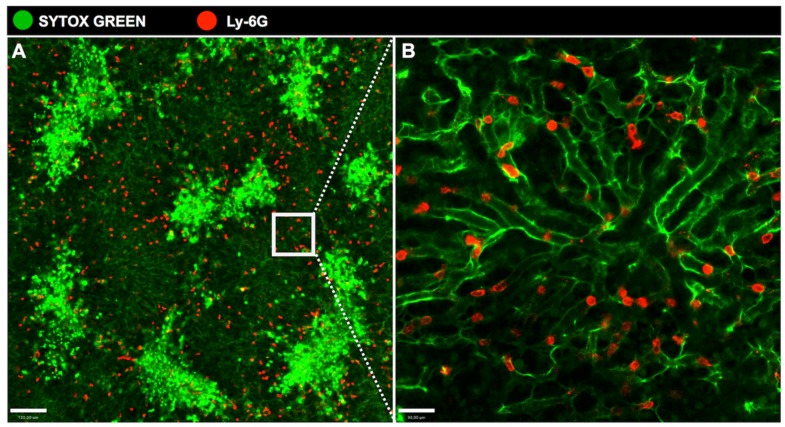
**Accumulation of neutrophils in the hepatic sinusoids during acute liver injury.** (**A**) Overdose of acetaminophen resulted in acute liver injury. Neutrophils, here stained with anti-LyCG PE (in red), are attracted to lesion sites in the liver, characterized by necrotic areas due to hepatocytes’ death, and stained with Sytox (bright green areas); (**B**) A higher resolution showing the neutrophils within the sinusoids. Scale bar in (**A**), 120 μm. Scale bar in (**B**), 30 μm. All images were acquired using an inverted Nikon Eclipse Ti coupled to an A1 scanning head with no modifications. All animal studies were approved by the Animal Care and Use Committee at Universidade Federal de Minas Gerais, Brazil (CEUA 147/2016).

Neutrophils, a subset of polymorphonuclear leukocyte, are the predominant immune cell population in human blood, being crucial for controlling bacterial and fungal infections. In healthy individuals, more than 10^9^ neutrophils per kg body weight are released from the bone marrow every 24 h [114]. Neutrophils develop from hematopoietic stem cells in bone marrow, a process called ‘granulopoiesis’, and granulocyte colony-stimulating factor (G-CSF) is the major factor regulating the neutrophil life cycle by increasing cell proliferation, survival, differentiation, and mobilization to blood circulation. In the liver, neutrophils migrate from the blood to the inflammatory focus driven by chemokynes and chemotactic agents. These mediators are released to establish an efficient chemotactic gradient within the liver intravascular compartment [115]. Once attracted, neutrophils accumulate within the hepatic microvasculature, which includes sinusoids and postsinusoidal venules, before transmigration process (Figure 5). Neutrophil transmigration involves the upregulation of adhesion molecules, such as selectins and transient interactions between selectins and their ligands result in neutrophil adhesion and rolling, the first step of the leukocyte recruitment cascade [116,117]. Neutrophil extravasation from the sinusoids into the parenchyma is mediated by β2 integrin, intercellular adhesion molecule-1 (ICAM-1), or β1 integrin/vascular adhesion molecule-1 (VCAM-1) interactions [118]. On the other hand, in situations of extensive endothelial cell damage, neutrophils may have direct access to the parenchyma without a CAM-dependent transmigration process [119]. Once at the inflammation site, neutrophils initiate clearance process, including phagocytosis, release of DNA extracellular traps. In addition, neutrophils can secrete a large amount of granules containing proteolytic enzymes (elastase, cathepsins and proteinase-3), bactericidal proteins (presenilin 1, defensins, bactericidal/permeability increasing protein), matrix metalloproteinases, and lysozymes [120,121].

Although neutrophils are known for their excellence in capturing and killing bacteria, these cells also play a key role in sterile liver damage [122]. Using a model of thermal hepatic injury, Wang and colleagues [123] demonstrated that neutrophils penetrate the injury site and perform the critical tasks of dismantling injured vessels and creating channels for new vascular regrowth. In contrast to what is seen in cases of clearance of pathogens, they neither die at the injury site nor are phagocytized by macrophages. Instead, many of these neutrophils reenter the vasculature and have a preprogrammed journey passing through the lungs before entering the bone marrow, where they undergo apoptosis. There is a long time debate regarding the role of neutrophil-induced cell injury in the liver. During hepatic inflammation, neutrophils are recruited to the damage, and in high number, these cells can generate sufficient oxidative stress to kill hepatocytes [124,125,126]. Neutrophils produce ROS through the NADPH oxidase system, initiating toxicity. Furthermore, myeloperoxidase, an enzyme present in neutrophil granules and released upon activation, causes significant oxidative stress and protein dysfunction [124,127]. Despite the damages caused by neutrophil infiltration and activity, the role of these cells in tissue repair is indispensable. In sterile liver lesion, 12 h after injury, the neutrophils fill the areas that had been occupied by the collapsed sinusoids helping in clearance the area. In contrast, antibody-mediated depletion of neutrophils results in far more debris remaining in the injury site and a delay on tissue repair [123]. Therefore, despite neutrophils’ role in exacerbating tissue damage in acute inflammatory responses, these cells may be crucial in the resolution and repairing phase.

#### 3.2.2. Eosinophils

The role for eosinophils and their actual location within the liver is still poorly understood. Eosinophils are granulocytes characterized by cytoplasmic granules with an affinity for acid aniline dyes, such as eosin. The origin of the eosinophils is also in the bone marrow, from pluripotent stem cells differentiated after stimuli of granulocyte-macrophage colony stimulating factor (GM-CSF), interleukin 3 (IL-3), and more particularly interleukin 5 (IL-5) [128]. Eosinophils are predominantly tissue cells that migrate from the blood into tissues as a result of several correlated events which involve adhesion pathways and chemoattractants [129]. The recruitment of eosinophils to the damaged liver is regulated by numerous events involving cytokines and chemokines released by another eosinophils and T lymphocytes [130]. Once at the damaged tissue, eosinophils may be activated by numerous mediators that can drive variable profiles of cell activation [131]. The location and the consequences of eosinophilic infiltration of the liver depends on the inflammation focus or disease condition. In biopsies from individuals with chronic hepatitis C the number of these cells was greater in the larger portal tracts and strongly associated with liver steatosis and fibrosis [132]. In a drug-induced liver injury (DILI), the role for eosinophil is controversial. Bjornsson and colleagues related a favorable outcome to the occurrence of liver eosinophilia after evaluation of 570 case reports of DILI [133]. In contrast, Pham et al. suggest that the release of cationic proteins by eosinophils may contribute to liver cell damage in patients with DILI based on immunohistochemical assays [131].

### 3.3. Lymphocytes

In humans, up to 65% of all hepatic lymphocytes consist of NK cells, NKT cells, and unconventional T cells (γδ) [134]. These cell populations can proliferate under certain experimental or pathological conditions. The dominating presence of these populations in the liver and in early defense against pathogens places these cells in a key position among effector lymphocytes in liver immune surveillance.

#### 3.3.1. Liver Natural Killer (NK) 

NK cells are classically known as a subset of the innate immune system, but can play an important role in shaping the adaptive immune response [135]. NK cells are also derived from the bone marrow and are distributed throughout the body in both lymphoid and non-lymphoid tissues [136,137]. However, a growing body of evidence has indicated that the presence of hematopoietic progenitors and immature NK cells at extra-medullary sites [138]. Interestingly, the liver is an NK cells enriched organ. Hepatic NK cells are not only a vast population in the liver, but are also naturally activated as they show higher cytotoxicity against tumor cells than other NK cells in rodents and in humans, including splenic or peripheral blood NK cells [139,140]. Over the last decade, data have suggested the involvement of NK cells in the pathogenesis of liver diseases, mainly tumors and viral infections [140,141,142,143].

In 1976, Wisse and colleagues described a new type of liver resident NK cells in rats, named “Pit cells” [144]. Further studies revealed that Pit cells are attached to the endothelial lining, which they might penetrate with the microvilli of parenchymal cells. These cells are often closely attached to KCs, suggesting some type of physical relationship [145]. The lobular distribution of Pit cells in the liver was found to be predominantly periportal (~60%). The human liver does not harbor a morphological equivalent of the rat Pit cell.

In mice, liver NK cells are present at significantly higher frequencies than NK cells in the bone marrow, peripheral blood, and spleen, accounting for approximately 5–10% of the total lymphocytes present in this tissue. Although liver-resident NK cells resemble immature circulating NK cells in phenotype, adoptive transfer studies showed that these cells preferentially home to the liver and do not convert to circulating phenotype, suggesting that liver-resident NK cells are stable under steady-state circumstances [138]. In humans, the liver also contains a unique CD49a^+^ NK cell subset that resembles murine liver-resident NK cells [146]. Similar to their counterparts in the mouse liver, human CD49a+ NK cells are T-bet^+^ Eomes^−^ and are not detectable in afferent or efferent hepatic venous or peripheral blood [145].

#### 3.3.2. Liver NKT Cells

NKT cells are a subset of lymphocytes that express both αβ TCR (T cell marker) and cell surface receptors characteristic of NK cells (NK1.1 in C57BL/6 mice) [147]. Mouse liver lymphocytes contain about 20% to 30% NKT cells, which are further elevated in pathological conditions. NKT cells play an important role in induction of liver injury in models of liver injury induced by concanavalin A, α-galactosylceramide, alcohol, and drugs [148]. To better understand the role of hepatic NKT cells, Geissman and colleagues observed in vivo that NKT cells patrol liver sinusoids to provide intravascular immune surveillance, and CXCR6 contributes to liver-based immune responses by regulating their abundance [149]. Besides, they observed that CXCR6-deficient mice exhibited a selective and severe reduction of CD1d-reactive NKT cells in the liver and decreased susceptibility to T-cell-dependent hepatitis. Therefore, it is believed that NKT cells are predominantly a population of intravascular cells.

#### 3.3.3. Liver γδ T Cells

Unconventional T cells that do not express NK cell markers include the major group of TCR γδ cells (also called γδ T cells). This group represents 15% to 25% of all intrahepatic T cells, thereby rendering the liver one of the richest sources of γδ T cells in the body [150]. γδ T cells have oligoclonal or invariant TCRs that recognize a limited range of antigens such as stress proteins and nonprotein antigens. In the liver, γδ T cells were predominantly found in portal infiltrates and areas of bile duct proliferation or fibrogenesis, but the exact contribution of these cells to liver immunopathology remained elusive [151]. However, the results obtained in human liver disease as well as murine models about the role of these cells are not fully conclusive at present, and the effects of γδ T cells on the outcome of liver disease might vary, depending on etiology and stage of disease.

The normal liver contains a large number of lymphocytes that include not only specialized NK and NKT cells, but also CD4 and CD8 T cells. In inflammatory conditions, the number of lymphocytes in the liver increases and the type and distribution of these infiltrating cells will determine the nature of the inflammation. Under healthy conditions, human liver contains significant numbers of T lymphocytes in the portal tracts and scattered through the parenchyma [152]. At homeostasis conditions of the liver, both CD4 and CD8 T cells are found in portal tracts albeit at low numbers and a population of cells with the characteristics of intraepithelial lymphocytes are found in association with biliary epithelium [153].

#### 3.3.4. Liver B Cells

B-lymphocytes perform various immunological functions, including production of antibodies, antigen presentation, secretion of multiple cytokines, and regulation of immune responses. However, little is known about the functional biology of liver B cells. The main reasons for the relative lack of knowledge in this regard may be due to the small number of B cells residing in the healthy liver and the experimental difficulty in isolating and analyzing specifically B cells [154]. Hepatic B cells comprise only ~5% of intrahepatic lymphocytes. During infection, intraportal lymphoid follicles display a germinal center-like structure in which activated B cells are surrounded by a follicular DC network. The distribution of IgM-, IgD-, and IgG-positive B cells and the gene expression patterns in intrahepatic germinal centers resemble those in lymph nodes, suggesting that intrahepatic germinal centers function as functional follicular structures [154,155].

## 4. Concluding Remarks 

It is becoming increasingly clear that the liver is not only an ‘accessory organ’ for the digestive system. Despite the vital role as a metabolic organ, the liver has emerged as one of the main immune and lymphoid organs of the body. In this context, the liver also harbors one of the most complex and active populations of immune cells in the body. New imaging and immunophenotyping techniques have allowed the identification of these different populations using both in vivo and in vitro assays (Table 1). Interestingly, these cells are extremely organized in the different liver compartments, and the hepatic hemodynamic scheme favors an intimate contact of blood contents with these cells. Therefore, expanding our knowledge on the frequency, activation status, and the changes in cell compartmentalization throughout the liver during diseases may hold interesting venues for investigation not only for basic science, but also for the ethiopathogenesis of different hepatic diseases.

## Figures and Tables

**Table 1:** Liver immune cells.

Liver Immune Cells	Location in Homeostasis	Main Surface Markers	Putative Role in Diseases
Kupffer Cells	Inside the sinusoids; adhered to the endothelium	F4/80, CD11b	Controlling inflammation; Kupffer cell depletion is associated with worse prognosis
Dendritic Cells	Underneath hepatic capsule; around large vessels	CD19^−^CD11c^+^; CD8α^+^B220^−^ CD11b^−^ (lymphoid); CD8α^−^ B220^−^CD11b^+^ (myeloid); B220^+^ CD11b^−^ (plasmacytoid)	Enhanced response to viral infections, controlling viral spread and T cell activation
Monocytes	Inside the sinusoids as patrolling cells	CD11b^hi^CD115^hi^Gr1^lo^	Infiltrating monocytes control pathogen spread and heal tissue injury
Neutrophils		Ly6G^+^CD11b^+^F4/80^−^	Overt infiltration is associated with enhanced liver injury in several models
Eosinophils		CD11b^+^CD193^+^Siglec F^+^	Role in pathogenesis of liver diseases through release of granules containing TNF-α, highly cytotoxic proteins such as major basic protein and eosinophilic cationic protein
Natural Killer Cells		CD3^−^NK1.1^+^	Involved in the pathogenesis of liver diseases, mainly tumors and viral infections; higher cytotoxicity than other NK cells
NKT Cells		CD3^+^NK1.1^+^	Patrolling liver sinusoids to provide intravascular immune surveillance
T lymphocytes		CD3^+^CD4^+^ (T CD4 cells); CD3^+^CD8^+^ (T CD8 cells)	Clearance of virus and in virus-induced immunopathology
B lymphocytes		CD19^+^	Antibody-secreting cells within germinal centers of intraportal lymphoid follicles, during viral hepatitis
γδ T cells		CD24^+^ CD25^−^ CD27^+^	Controlling early viral infections; expressing perforin, lysing virus-infected targets, and releasing IFN-γ
